# Exploring the biochemical and biological functions of copper radical oxidases in the vascular wilt phytopathogen *Verticillium dahliae*

**DOI:** 10.1128/aem.00790-26

**Published:** 2026-06-03

**Authors:** Jessica K. Fong, Ying-Yu Chen, Rebekka Harting, Moritz Klein, Simone Lewandowski, Yann Mathieu, Mireille Haon, Bastien Bissaro, Jean-Guy Berrin, Ivo Feussner, Gerhard H. Braus, Harry Brumer

**Affiliations:** 1 Michael Smith Laboratories, University of British Columbia 8166, Vancouver, BC, Canada; 2 Department of Chemistry, University of British Columbia 8166, Vancouver, BC, Canada; 3 Institute of Microbiology and Genetics, Georg-August-Universität Göttingen 9375, Göttingen, Germany; 4 Göttingen Center for Molecular Biosciences (GZMB), Georg-August-Universität Göttingen 9375, Göttingen, Germany; 5 Department of Plant Biochemistry, Albrecht-von-Haller-Institute for Plant Sciences, Georg-August-Universität Göttingen 9375, Göttingen, Germany; 6 INRAE, Aix Marseille University, Biodiversité et Biotechnologie Fongiques (BBF), Marseille, France; 7 Service Unit for Metabolomics and Lipidomics, Göttingen Center for Molecular Biosciences (GZMB), Georg-August-Universität Göttingen 9375, Göttingen, Germany; 8 Department of Botany (Associate Member), University of British Columbia 8166, Vancouver, BC, Canada; The University of Arizona, Tucson, Arizona, USA

**Keywords:** reverse genetics, enzymology, *Verticillium*, glyoxal oxidase, galactose oxidase, copper radical oxidase

## Abstract

Plant pathogenic fungi pose a significant threat to global food security. Hence, discovering targeted (bio)chemical methods to combat plant diseases is a critical research avenue. Recent work has implicated copper radical oxidases (CROs) from Auxiliary Activity Family 5 (AA5) in fungal morphogenesis and pathogenesis, including in appressorium penetration by foliar phytopathogens in the genera *Colletotrichum* and *Magnaporthe*. We identified orthologous AA5-encoding genes in *Verticillium* species; however, it was not clear that the functions of the corresponding CROs would be conserved in these vascular wilt pathogens. Using *Verticillium dahliae* JR2 as an exemplar, recombinant protein production and detailed enzyme kinetic analyses revealed predominant aryl-alcohol oxidase activity of the AA5 subfamily 2 (AA5_2) homolog, and predominant aldehyde oxidase activity of the AA5 subfamily 1 (AA5_1) homolog. Transcriptomics and reverse genetics experiments on both CROs and three putative peroxidases/catalases (known activators of CROs) in *V. dahliae* JR2 failed to indicate direct roles in phytopathogenesis. Taken together, the data indicate that the biological roles of CROs and AA5_2 members, in particular, are not broadly conserved between foliar and vascular wilt phytopathogens. However, the biochemical characterization of an AA5_1 aldehyde oxidase and an AA5_2 aryl-alcohol oxidase from *V. dahliae*, as well as an AA5_2 ortholog from *V. longisporum* VL43, provides a foundation for further functional elucidation, as well as new biocatalysts for biotechnological applications.

**IMPORTANCE:**

Plant pathogens constitute a considerable burden to human society by attacking crops and reducing agricultural yields. Oxidative enzymes are often key weapons in the arsenal deployed by phytopathogens in the effort to breach cell walls and extract nutrients. Here, the characterization of the biochemical specificities of copper radical oxidases (CROs) from Verticillium wilt/stripe fungi defines a range of possible alcohol and aldehyde substrates *in vivo* and outlines the potential of these enzymes for their biotechnological application for chemical valorization. Although specific gene knockouts did not reveal a biological function for these CROs, we now know that the molecular mechanism of CRO-mediated pathogenesis previously observed in foliar phytopathogens from the genera *Colletotrichum* and *Magnaporthe* is not conserved in the vascular wilt pathogen *Verticillium*.

## INTRODUCTION

Fungal phytopathogens threaten agricultural production and global food security. The annual losses of crop yield due to plant diseases are estimated to be over USD $220 billion (1–3). Some of the most devastating phytopathogens belong to the *Verticillium* species complex, which cause vascular wilts (Verticillium wilt/stripe) in a diverse range of plant species. Of the 10 defined *Verticillium* species, *Verticillium dahliae* is an agriculturally important species due to its broad adaptation to over 200 host species from various plant families, which include important crops such as cotton, canola, potatoes, and tomatoes (4–8).

The disease cycle of *V. dahliae* begins with the germination of dormant microsclerotia, which are robust melanized structures formed by many fungal pathogens to survive long periods of hostile environmental conditions. The germination of microsclerotia is usually initiated when certain stimuli (e.g., root exudates) and hospitable conditions (e.g., temperature, pH, moisture, and susceptible hosts) are encountered (9, 10). *V. dahliae* infects plant hosts through the penetration of the host root system by the fungal hyphae. This process is aided by the formation of infection structures called hyphopodia that can further develop into penetration pegs (11). Once the root system has been colonized, *V. dahliae* enters the xylem and propagates through the host vascular system, causing a systemic infection and clogging the vessels, which results in disease symptoms such as wilting, tissue chlorosis, etc. As the host tissues degrade, *V. dahliae* transitions to a saprophytic phase, where foliar tissues are also colonized, and microsclerotia are produced for release into the soil after death and decomposition of the plant host (4, 12). The microsclerotia remain viable for long periods of time in the absence of host plants. Such prolonged survival of a fungus in the form of resting structures is a common strategy in several other phytopathogenic fungi, for example, the eponymous *Sclerotinia sclerotiorum*, the causal agent of white stem rot (13).

Current efforts to combat Verticillium wilt include crop rotation, resistant cultivar breeding, chemical fungicides, and biocontrol—to name a few. However, none are completely effective, as Verticillium wilt continues to cause extensive economic crop losses (14). A targeted method of controlling Verticillium wilt may lie within the biochemical mechanisms that *V. dahliae* employs during host infection. Phytopathogenic fungi employ a diverse arsenal of cell-wall-degrading enzymes (CWDEs) to breach this crucial physical barrier against pathogen attack (15). For instance, the genome of *V. dahliae* encodes 489 predicted carbohydrate-active enzymes (CAZymes) (16), which comprise diverse families of hydrolases, lyases, and oxidoreductases (17, 18). Within the CAZy classification, Auxiliary Activity Family 5 (AA5) comprises copper radical oxidases (CROs) that are further classified into two phylogenetic subfamilies, AA5 subfamily 1 (AA5_1) and AA5 subfamily 2 (AA5_2), with primary activities on aldehydes (e.g., methylglyoxal) and alcohols (e.g., galactosides and alkanols), respectively (17, 19). CROs were originally postulated to participate in biomass degradation through the production of hydrogen peroxide as a co-substrate for ligninolytic enzymes (20, 21). However, several studies have recently also implicated CROs in fungal (and bacterial) morphogenesis and virulence (reviewed in reference 22).

In particular, a recent study of foliar phytopathogenic fungi from the genera *Colletotrichum* and *Magnaporthe* uncovered a tandem arrangement of conserved genes that encodes an AA5_2 CRO/heme-peroxidase enzyme pair, which is co-secreted during early infection at specialized penetration structures known as appressoria (23, 24). Biochemical studies demonstrated that the CRO is a general alcohol oxidase (AlcOx) and that the partner peroxidase is required for catalytic activation of the AlcOx (25). The pair are essential to effect plant-cell entry by oxidizing long-chain aliphatic alcohols found in the waxy leaf cuticle, possibly involving a signaling cascade (23, 24). However, it is unknown if a similar system is conserved across phytopathogenic fungi, such as *Verticillium* species, which employ a different mode of infection (root vs. aerial tissue) and do not form appressoria.

Here, a combination of biochemical and reverse-genetic analyses was used to probe the *in vitro* activity and potential roles of AA5_1 and AA5_2 CROs in *Verticillium* pathogenesis, using *V. dahliae* as a model. The biochemical characterization of select homologs from *V. longisporum* was also performed. The results detail the substrate range of CROs within these *Verticillium* species and also demonstrate that the physiological roles of CROs are not conserved across fungal phytopathogens.

## RESULTS

### Bioinformatic analysis

**Fig 1:**
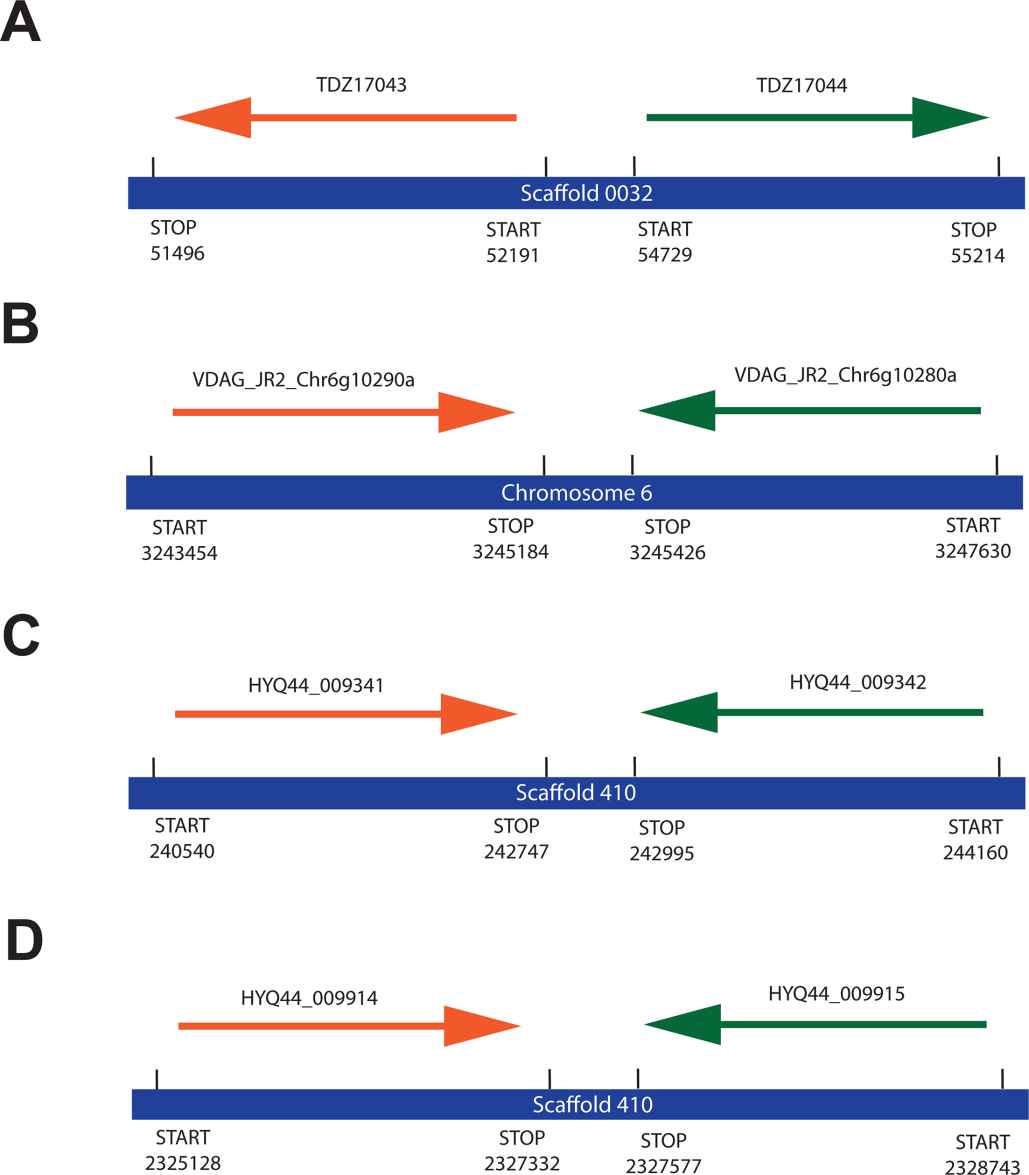
Genomic localization and orientation of genes encoding AA5_2 and catalase enzymes. (**A**) *C. orbiculare.* (**B**) *V. dahliae* JR2. (**C and D**) *V. longisporum* VL43. Genes encoding AA5_2 and catalase enzymes are denoted as orange and green arrows, respectively. GenBank or Ensembl Fungi accession codes are indicated in text above the arrows.

Analysis of the genome of *V. dahliae* JR2 (haploid species) (26) indicated the presence of one AA5_1 (Ensembl Fungi: VDAG_JR2_Chr3g06330a) and one AA5_2 member (VDAG_JR2_Chr6g10290a). Interestingly, the AA5_2 gene, which encodes a protein 45% identical to the AlcOx (AA5_2) in *Colletotrichum orbiculare*, was found in a tail-to-tail arrangement with a neighboring gene predicted to encode a mono-functional heme catalase (VDAG_JR2_Chr6g10280a), which shares common features with peroxidases (27). In contrast, the AlcOx gene in *C. orbiculare* is found in a head-to-head arrangement in tandem with a heme-peroxidase (Fig. 1) (24). The genome of *V. dahliae* JR2 also encodes genes for two enzymes annotated as heme-peroxidases, *Vda*PerOx1 (VDAG_JR2_Chr5g05870a) and *Vda*PerOx2 (VDAG_JR2_Chr2g10970a), that share 40% protein sequence identity to those in *Colletotrichum* species. However, these genes are located on a different chromosome from the AA5_2 and catalase-encoding genes.

*V. longisporum* VL43, a diploid species (26), correspondingly contains genes encoding two AA5_1 (GenBank: HYQ44_011208, HYQ44_012414) and two AA5_2 members (HYQ44_009914, HYQ44_009341). Similar to *V. dahliae* JR2*,* both AA5_2 genes in *V. longisporum* VL43, which share 43% sequence identity to *Cor*AlcOx, are found in a tail-to-tail arrangement with a neighboring catalase gene (HYQ44_009915, HYQ44_009342) (Fig. 1C and D). Like *V. dahliae* JR2, the genome of *V. longisporum* VL43 contains two genes encoding peroxidases (HYQ44_016687, HYQ44_003627). These share 70% sequence identity with the putative heme-peroxidases from *V. dahliae* JR2 and 35% sequence identity with the *C. orbiculare* peroxidase.

Due to the tandem genomic colocalization of the AA5_2 members and the catalases in *Verticillium* species, we hypothesized that they might play homologous roles to those of the AlcOx-PerOx pair found in the foliar phytopathogens, *Colletotrichum spp*. and *Magnaporthe spp.* (23, 24). In these organisms, the AlcOx/PerOx pairs are co-secreted (24). SignalP analysis of the *V. dahliae* JR2 and *V. longisporum* VL43 AA5_2 amino acid sequences indicates that these enzymes likewise contain secretion signal peptides. The respective neighboring putative catalase genes, however, do not encode signal peptides, suggesting either intracellular localization or the possibility of an unconventional secretion pathway. In contrast, the putative peroxidases encoded elsewhere in the *V. dahliae* JR2 and *V. longisporum* VL43 genomes do contain signal peptides, implying canonical secretion. Interestingly, the single *V. dahliae* JR2 AA5_1 member has a predicted signal peptide for extracellular secretion, while only one of the two AA5_1 members from *V. longisporum* VL43 (HYQ44_011208) was predicted to be secreted.

**Fig 2:**
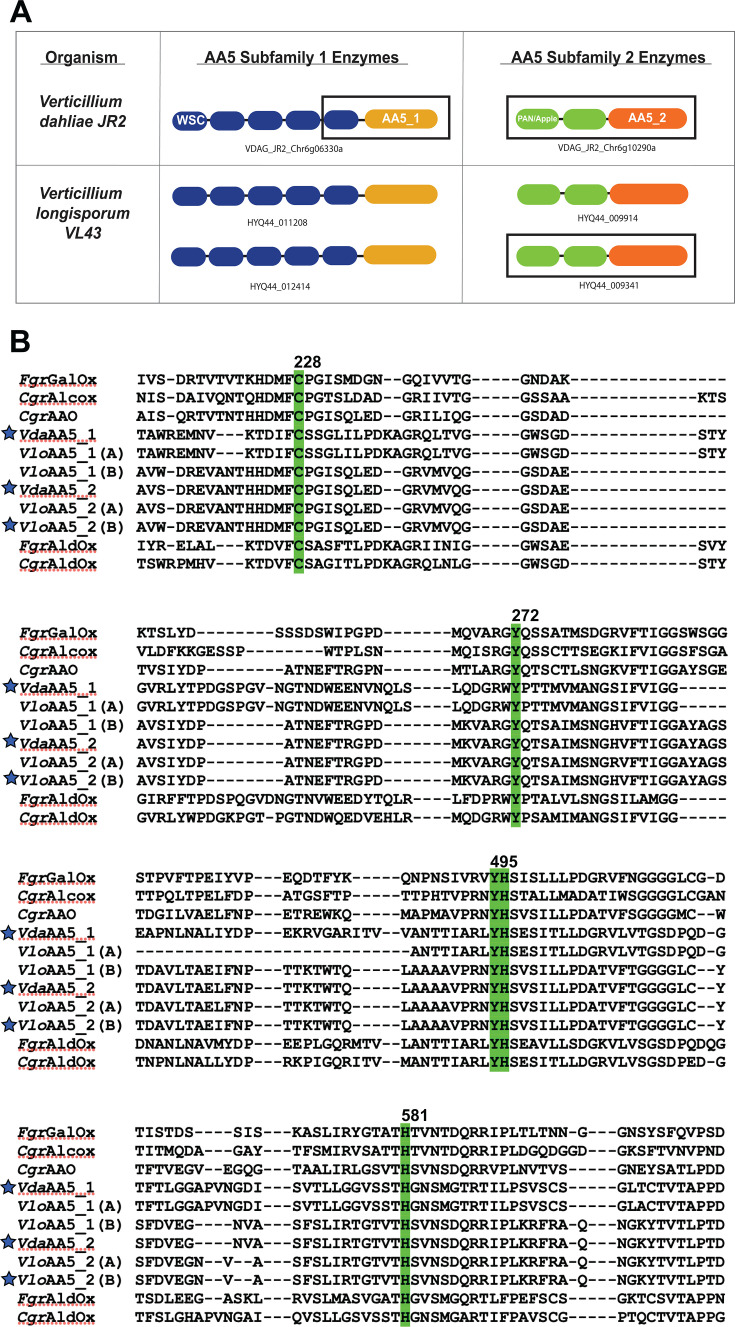
Enzyme modularity and conservation of catalytic residues in AA5 enzyme candidates. (**A**) Modularity of AA5_1 and AA5_2 enzymes from *V. dahliae* JR2 and *V. longisporum* VL43. Wall stress-responsive component (WSC) domains, PAN/Apple, AA5_1, and AA5_2 catalytic domains are colored in blue, green, yellow, and orange, respectively. Genomic locus tags for each enzyme are provided in the main text. Boxes denote the protein constructs that were successfully cloned and expressed. (**B**) Multiple sequence alignment of *Verticillium* AA5 sequences with reported AA5 enzymes. Conserved AA5 catalytic residues highlighted in green and numbered according to the sequence of the archetypal *Fusarium graminearum* galactose oxidase (*Fgr*GalOx). *Verticillium* AA5 enzymes successfully produced recombinantly in this work are denoted with blue stars. *Fgr* = *Fusarium graminearum*, *Cgr* = *Colletotrichum graminicola*, *Vda* = *Verticillium dahliae*, *Vlo* = *V. longisporum*, GalOx = galactose oxidase, AlcOx = alcohol oxidase, AAO = aryl alcohol oxidase, and AldOx = aldehyde oxidase.

Further sequence analysis of the *V. dahliae* and *V. longisporum* AA5_2 enzymes indicated the presence of two N-terminal PAN/Apple domains (Fig. 2A). The AA5_1 members of *V. dahliae* JR2 and *V. longisporum* VL43 contain five N-terminal WSC (wall stress-responsive component) domains (Fig. 2A). PAN/Apple and WSC domains are widely found in AA5 CROs, although their functions are largely unresolved (28). Additionally, multiple sequence alignments of the putative AA5 members from *V. dahliae* and *V. longisporum,* along with other reported AA5 enzymes, showed the presence of catalytic residues that are highly conserved within all AA5 members (Fig. 2B).

### Recombinant enzyme production

As a first step to understanding the roles of AA5 CROs in *Verticillium* species, genes encoding the AA5_1 member, the AA5_2 member, and the three putative catalase/heme-peroxidases from the model strain *V. dahliae* JR2 were chosen for recombinant expression and subsequent biochemical characterization. To further validate substrate specificity and enable direct comparison, the genes encoding the two AA5_2 members and their putative catalases from *V. longisporum* VL43 were also selected.

cDNA sequences encoding the full-length proteins of the single AA5_2 enzyme from *V. dahliae* JR2 and both AA5_2 enzymes from *V. longisporum* VL43, excluding the predicted native signal peptides, were commercially synthesized and cloned into *Komagataella phaffii* (syn. *Pichia pastoris*) using the pPICZαA vector containing the *Saccharomyces cerevisiae* alpha-factor secretion signal peptide. The *V. dahliae* AA5_2 enzyme was successfully produced as a stable, secreted protein. However, only one of the AA5_2 paralogs from *V. longisporum* VL43, encoded by HYQ44_009341, was recombinantly produced (Fig. 2A).

The commercial synthesis of the full-length cDNA encoding the *V. dahliae* AA5_1 member was unsuccessful, likely due to sequence complexity arising from the multiple WSC domain repeats. Instead, a truncated construct encoding a single WSC module and the AA5_1 catalytic module (Fig. 2A) was successfully cloned into *K. phaffii* and expressed, yielding a secreted protein.

The same approach was carried out to obtain recombinant *K. phaffii* strains harboring the putative catalases and peroxidases from *V. dahliae* JR2 and *V. longisporum* VL43. Unfortunately, we were unsuccessful in obtaining recombinant forms of these proteins, despite our previous experience with the *Colletotrichum* and *Magnaporthe* homologs (23, 24).

### Activity and substrate specificity of *V. dahliae* AA5 CROs

**Fig 3:**
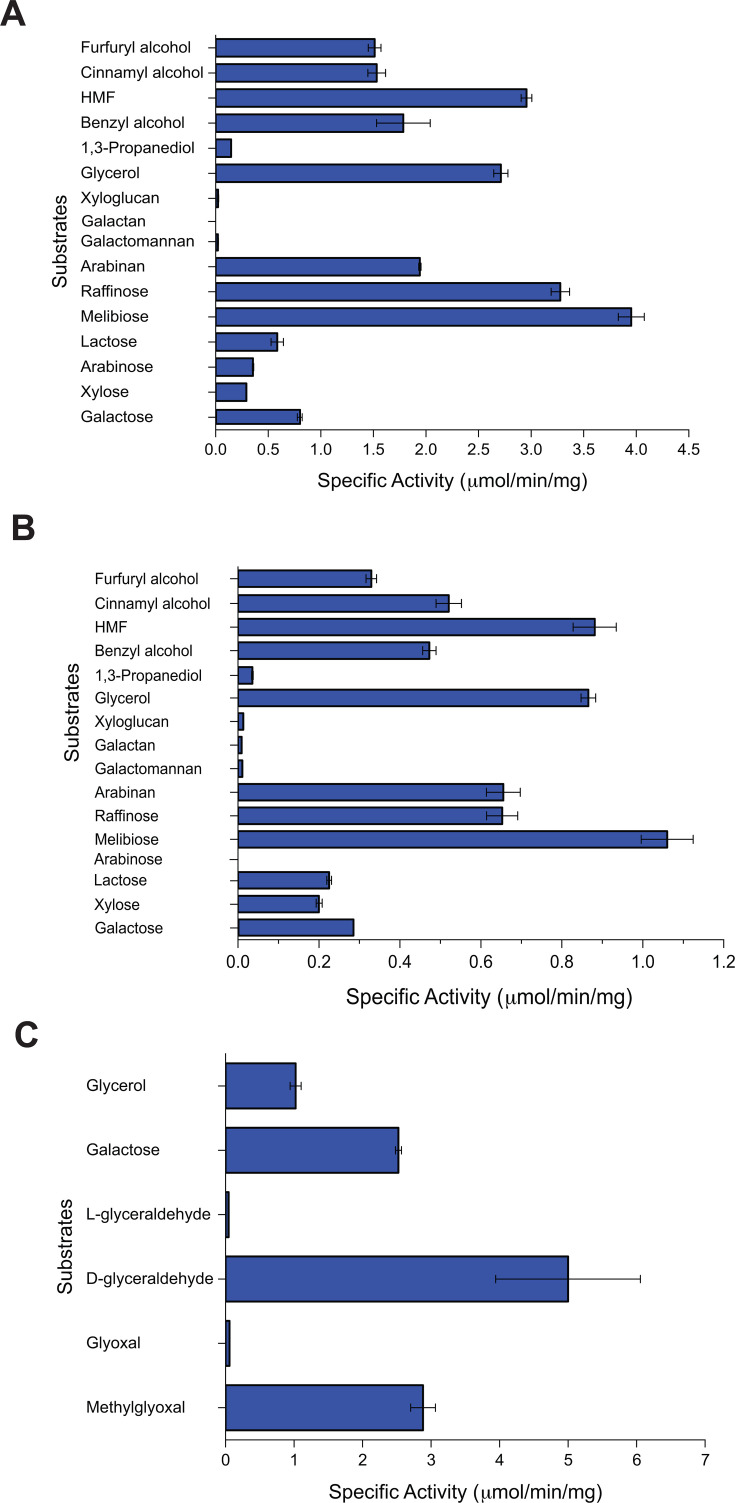
Specific activities of *V. dahliae* and *V. longisporum* AA5 enzymes on a panel of representative substrates (**A**) *Vda*AA5_2, (**B**) *Vlo*AA5_2, and (**C**) *Vda*AA5_1. Specific activity was determined using the HRP-ABTS coupled assay with 50 mM sodium phosphate buffer, pH 7.0 at room temperature, using 300 mM carbohydrates, 300 mM glycerol, and 10 mM alcohols and aldehydes. Bars show average values, and error bars show standard deviations over triplicate measurements. Individual-specific activity values are presented in Table S1.

The AA5_2 members of *V. dahliae* JR2 and *V. longisporum* VL43 were first tested against a panel of substrates that are typical of CROs in this subfamily (galactosides, other aliphatic alcohols, and aryl alcohols; Fig. 3A and B and Table S1), at room temperature and pH 7.0. *Vda*AA5_2 and *Vlo*AA5_2 had similar activity profiles, although *Vda*AA5_2 was 3-fold more active than *Vlo*AA5_2 on the same substrates. The highest specific activities were for carbohydrates (e.g., melibiose and raffinose) and aromatic alcohols (e.g., hydroxymethylfurfural [HMF] and cinnamyl alcohol). Interestingly, *Vda*AA5_2 and *Vlo*AA5_2 showed little to no activity on the aliphatic alcohols tested except for glycerol, which was later shown to be a poor substrate (Table 1). Subsequently, melibiose was chosen to determine pH-rate profiles. *Vda*AA5_2 and *Vlo*AA5_2 had optimal activities at pH 6.0 (Fig. S1A and S1B).

**TABLE 1:** Michaelis-Menten parameters for selected substrates

Enzyme	Substrate	*K*_M_ (mM)	*k*_cat_ (s^−1^)	*k*_cat_*/K*_M_ (M^−1^s^−1^)^*a*^	pH	Temperature (˚C)
*Vda*AA5_2	Hydroxymethyl furfural	120 ± 40	190 ± 60	1,580 (1,400)	6.0	25
	Cinnamyl alcohol	77 ± 20	40 ± 9	520 (530)		
	Glycerol	1,990 ± 380	45 ± 8	23 (19)		
	Raffinose	n.a.^*b*^	n.a.	(31)		
	Melibiose	n.a.	n.a.	(23)		
	Galactose	n.a.	n.a.	(6)		
*Vlo*AA5_2	Hydroxymethyl furfural	38 ± 10	13 ± 3	350 (300)	6.0	25
	Cinnamyl alcohol	n.a.	n.a.	(90)		
	Glycerol	150 ± 13	1.5 ± 0.1	9 (7)		
	Raffinose	n.a.	n.a.	(8)		
	Melibiose	115 ± 12	1.2 ± 0.1	10 (8)		
	Galactose	n.a.	n.a.	(2)		
*Vda*AA5_1	D-glyceraldehyde	132 ± 16	155 ± 17	1,180 (1,044)	7.0	25
	Methylglyoxal	44 ± 3	19 ± 0.8	430 (350)		
	Galactose	n.a.	n.a.	(15)		
	Glycerol	n.a.	n.a.	(8)		

^*a*^ Values in parentheses correspond to k_cat_*/K*_M_ values determined from the slopes of linear fits to initial-rate kinetic data substrate concentrations well below saturation; individual *K*_M_ and *k*_cat_ values were not calculated.

^*b*^ n.a., not applicable.

Further analysis of the substrate preferences of *Vda*AA5_2 and *Vlo*AA5_2 was conducted through initial-rate kinetics measurements on a selection of key substrates (Figs. S2 and S3). Optimal growth temperatures for *Verticillium* species can range between 20˚C and 27˚C (29); hence, these kinetics measurements were carried out at 25˚C. As summarized in Table 1, the highest catalytic efficiencies for *Vda*AA5_2 and *Vlo*AA5_2 were seen on aromatic alcohols such as HMF and cinnamyl alcohol. For both enzymes, the *k*_cat_*/K*_M_ values for HMF were three times that of cinnamyl alcohol. Commensurate with specific activity measurements, *Vda*AA5_2 is 3-fold to 5-fold more active on the same substrates when compared to *Vlo*AA5_2. Compared to cinnamyl alcohol and HMF, the catalytic efficiencies for glycerol and carbohydrates (galactose, melibiose, and raffinose) were significantly lower, *viz*., around 1–3 orders of magnitude lower depending on the substrate. These results are similar to those for other CROs that have been defined as aryl alcohol oxidases (EC 1.1.3.7), for example, *Cgr*AAO (30), *Fgr*AAO (31), and *Ast*AAO (32).

The specific activities of *Vda*AA5_1 were first determined on a small panel of common AA5_1 substrates, primarily aldehydes, at room temperature and pH 7.0. Due to the high activity of *Vda*AA5_1 on methylglyoxal, it was subsequently chosen as the substrate for pH-rate profile determination (Fig. 3C and Table S1). The optimum pH for *Vda*AA5_1 was pH 7.0 (Fig. S1C). At this pH, *Vda*AA5_1 displayed a similar activity profile to previously characterized AA5_1 enzymes (33–39), in which the highest specific activities were observed on small aldehydes such as methylglyoxal and glyceraldehyde. *Vda*AA5_1 stereoselectively oxidized d-glyceraldehyde rather than the l-isomer (Fig. 3C).

To refine the specificity analysis, initial rate kinetics were also obtained for select substrates at the optimal pH and 25˚C (Fig. S4 and Table 1). *Vda*AA5_1 had the highest substrate specificity for d-glyceraldehyde, followed by methylglyoxal, for which *k*_cat_/*K*_M_ was 2-fold lower. (Table 1). Mirroring the specific activity measurements above, very poor activity was observed on l-glyceraldehyde. The preference of *Vda*AA5_1 for aldehydes is further underscored by the catalytic efficiencies for glycerol and galactose, which are more than 70 times and 140 times lower, respectively, compared to d-glyceraldehyde. These results, which are similar to those of other AA5_1 members in the literature, collectively indicate that there is little variation of substrate preference among AA5_1 members (33–40), unlike AA5_2 enzymes, which are active on a range of carbohydrate, alkyl, and aromatic alcohol substrates (32, 41).

### *Ex vivo* metabolomics

Due to the broad range of substrates oxidized by AA5 enzymes, the identity of their natural substrate(s) remains elusive. In an attempt to identify putative physiological substrates, *Vda*AA5_2, *Vlo*AA5_2, and *Vda*AA5_1 were added to soluble extracts from *Arabidopsis thaliana* roots, followed by *ex vivo* metabolite fingerprinting via liquid chromatography-tandem mass spectrometry (LC-MS/MS) analysis. Unfortunately, the results obtained were inconclusive: Initial hits to lipids, dipeptides, and flavonoids based on parent ions could not be verified by fragmentation and comparison with available reference spectra.

### Transcriptomic analysis of *V. dahliae* genes

**Fig 4:**
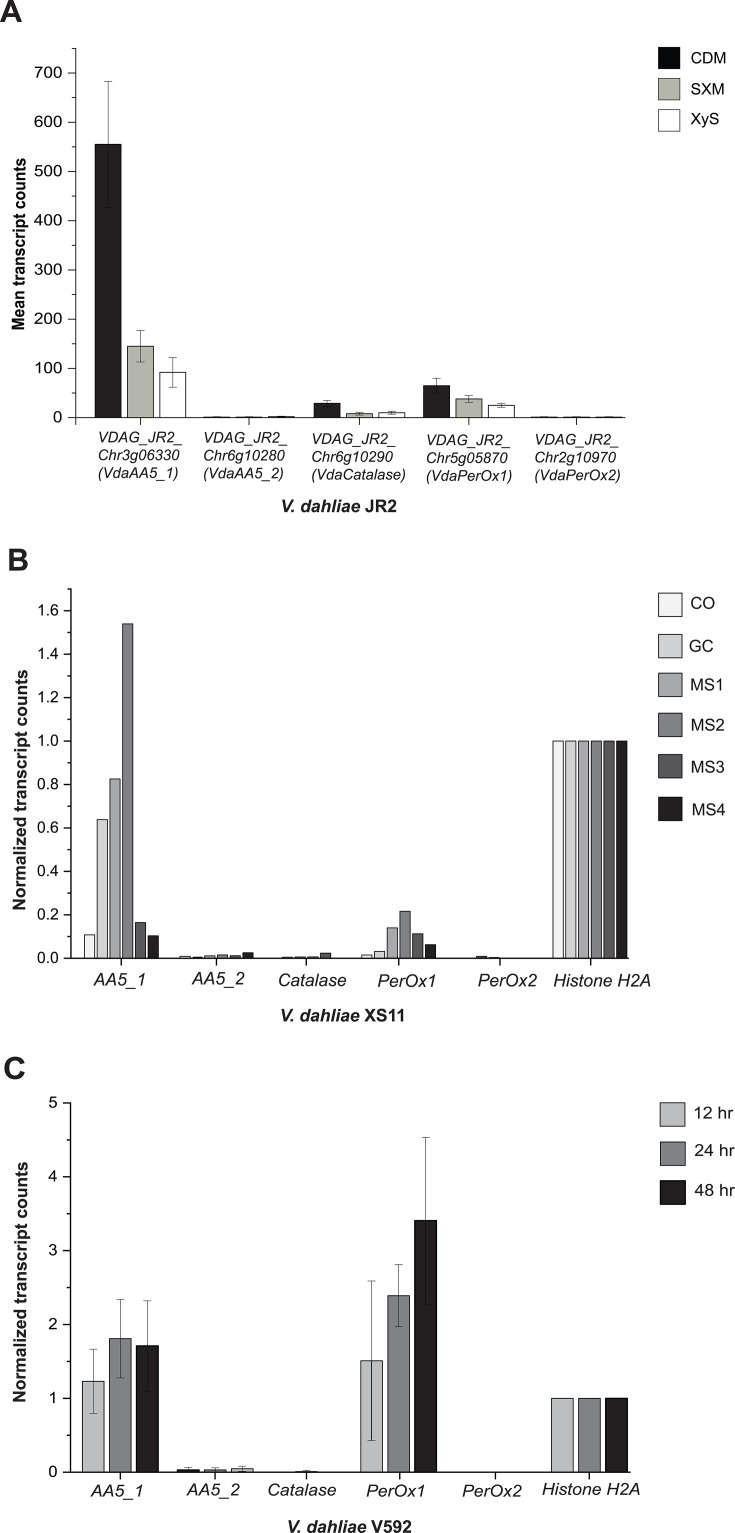
Transcription patterns of different *V. dahliae* strains. (**A**) *V. dahliae* JR2 candidate gene expression in control Czapek Dox medium (CDM) before switching to growth in simulated xylem media (SXM) and extracted tomato xylem sap (XyS). (**B**) Transcription profile of *V. dahliae* strain XS11 during microsclerotia development when grown on potato dextrose agar. CO = conidia, GC = conidia germination, MS1 = microsclerotia formation (60 h), MS2 = microsclerotia formation (72 h), MS3 = microsclerotia formation (96 h), and MS4 = microsclerotia formation (14 days). (**C**) Transcription profile of *V. dahliae* strain V592 after 12, 24, and 48 h post-inoculation of host (cotton). Transcript counts for panels B and C were normalized to the levels of the *Histone H2A* housekeeping gene. Transcript counts were obtained from public RNA-seq data sets accessible on the NCBI BioProject database (A: SXM: PRJNA1346639, XyS: PRJNA1364727, B: PRJNA208120, and C: PRJNA1219802).

Analysis of RNA-seq datasets of *V. dahliae* JR2 grown in Czapek Dox medium (CDM) before switching to simulated xylem medium (SXM) (BioProject: PRJNA1346639) or extracted tomato xylem sap (XyS) (PRJNA1364727), indicated that the *Vda*AA5_2 gene was transcribed at a very low level, regardless of medium composition (Fig. 4A). Transcripts of the neighboring catalase gene and the putative extracellular peroxidases were also generally not strongly nor differentially expressed. In contrast, the gene encoding *Vda*AA5_1 was notably transcribed in CDM, but curiously was downregulated ca. 4-fold and 5-fold in SXM and XyS, respectively. Overall, the transcript analysis failed to provide a compelling indication of the response of the CRO and catalase/peroxidase genes to plant material.

However, as the SXM and XyS media mimic plant material and may not capture the true *in planta* expression, other public RNA-seq data sets of *V. dahliae* strains were analyzed. In the transcription profile of *V. dahliae* XS11 (PRJNA208120) grown on potato dextrose agar, expression of the AA5_1 gene was observed during conidial germination and upregulation was observed during microsclerotia formation after 72 h, after which the gene was significantly downregulated in comparison to a housekeeping gene—Histone *H2A* (Fig. 4B). The gene encoding PerOx1 was also seen to be transcribed, albeit at lowered levels, while the AA5_2, catalase, and PerOx2 genes were not transcribed *in planta* during microsclerotia formation (42).

Similarly, analysis of a public RNA-seq data set from a recent study of wild-type cotton plants infected with *V. dahliae* V592 (PRJNA1219802) analyzed at 12, 24, and 48 h post-inoculation revealed that the genes encoding the AA5_1 enzyme and PerOx1 were upregulated in comparison to the Histone *H2A* gene (Fig. 4C) (43). In this case, the genes encoding the AA5_2 enzyme, catalase, and PerOx2 were also neither strongly nor differentially expressed.

### Reverse genetic analysis

Gene knockout experiments were carried out in *V. dahliae* JR2 to further explore the biological roles of these redox enzymes. Deletion cassettes containing a hygromycin or nourseothricin marker in place of the *V. dahliae* genes encoding *Vda*AA5_2, *Vda*AA5_1, catalase, PerOx1, and PerOx2 were generated and transformed into *Agrobacterium tumefaciens* for transfer into *V. dahliae* JR2. Δ*Vda*AA5_2, Δ*Vda*PerOx1, and Δ*Vda*AA5_1 deletion mutants were successfully generated and confirmed via Southern blot analysis (Fig. S5). We were unable to obtain the deletion mutants Δ*Vda*Catalase and Δ*Vda*PerOx2, despite repeated efforts. Initially, Δ*Vda*AA5_1, Δ*Vda*AA5_2, and Δ*Vda*PerOx1 were tested in several osmotic and oxidative stress conditions; however, no apparent differences from the wild-type were observed (Fig. S6 through S8).

**Fig 5:**
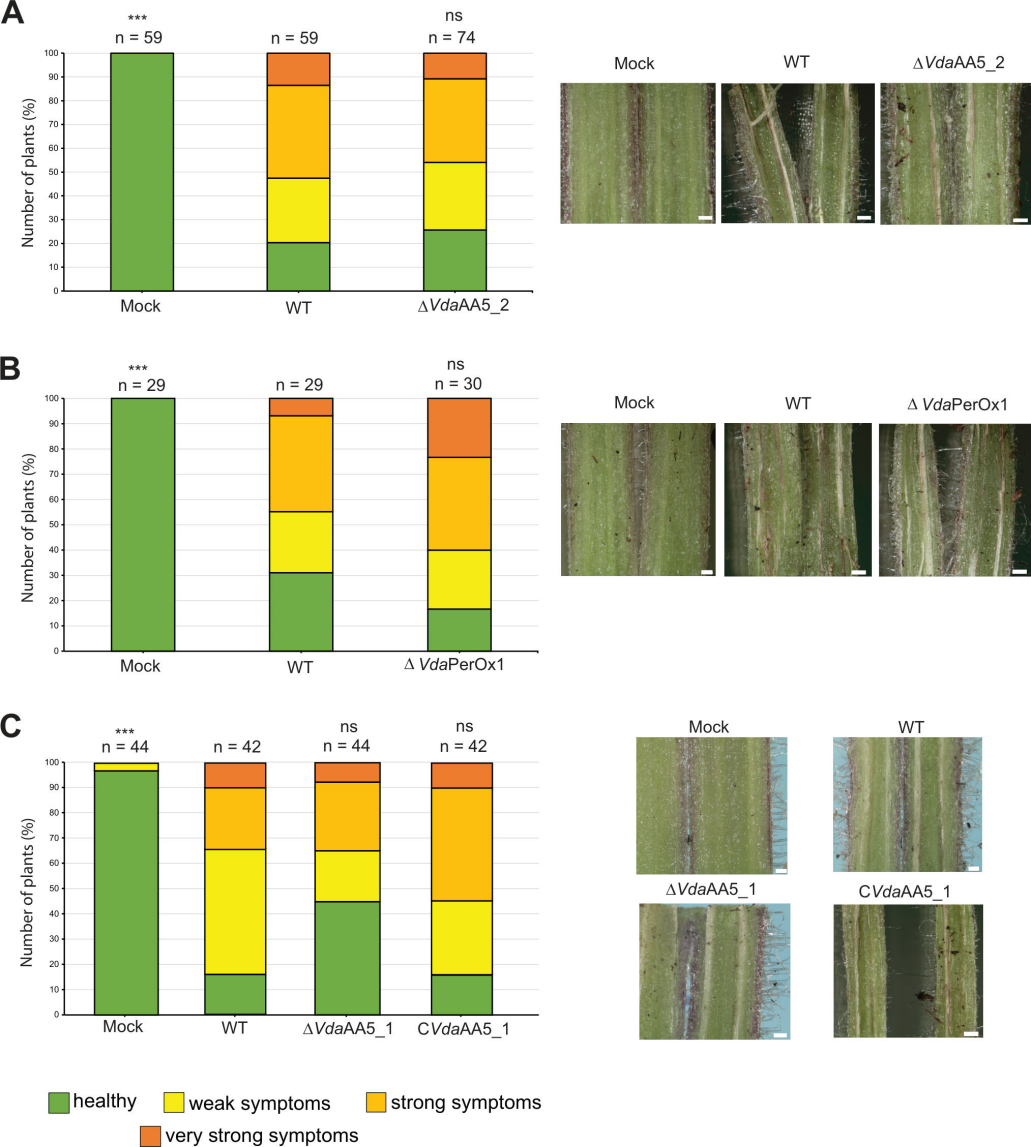
*In planta* assessment of the virulence effects of *Vda*AA5_2, *Vda*PerOx1, and *Vda*AA5_1. (**A**) Infection with the Δ*Vda*AA5_2 strain, (**B**) infection with the Δ*Vda*PerOx1 strain, and (**C**) infection with the Δ*Vda*AA5_1 and C*Vda*AA5_1 (complementation) strain. Ten-day-old tomato seedlings were infected by spores of JR2 wild-type (WT) or Δ*Vda*AA5_2, Δ*Vda*PerOx1, Δ*Vda*AA5_1, and C*Vda*AA5_1 strains individually via root dipping. Water-treated plants (mock) served as a control. Plants were incubated for 16:18 h (light:dark) at 22˚C–25˚C in a climate chamber. General disease scores (key shown at the bottom of the figure) were determined 21 days post-inoculation based on plant height, weight, and the length of the longest leaf. The sample sizes for each set of infections are noted above the bars (N); ≥15 biological replicates for each set were carried out. Statistical analysis was performed using the Mann-Whitney U test and indicated above the sample sizes as ***: *P* < 0.001 and ns = not significant.

Turning to studies *in planta*, tomato plants were inoculated, via root dipping, with the Δ*Vda*AA5_2, Δ*Vda*PerOx1, and Δ*Vda*AA5_1 strains individually. The wild-type strain and water were applied individually as positive and negative controls, respectively. No significant differences in disease symptoms were observed between plants infected with Δ*Vda*AA5_2 or Δ*Vda*PerOx1 and the wild-type strain (Fig. 5A and B). Plants infected with Δ*Vda*AA5_1 resulted in a larger proportion of healthy plants and a reduced proportion of plants with weak symptoms compared to the plants infected with wild-type strain (Fig. 5C). To validate the results seen with Δ*Vda*AA5_1, a *Vda*AA5_1 complementation strain (C*Vda*AA5_1) was generated and confirmed via Southern blot analysis (Fig. S5C). Plants infected with C*Vda*AA5_1 showed disease scores consistent with those of the wild-type strain (Fig. 5C). Despite this, the effects of *Vda*AA5_1 in *V. dahliae* virulence were not statistically significant (Fig. 5C).

## DISCUSSION

Plant pathogens pose a significant threat to the global food supply, decimating approximately 40% of annual crop yields (2). Therefore, uncovering the biochemical mechanisms employed by plant pathogens during their disease cycles can provide useful information for combating various plant diseases. Here, we provided a detailed biochemical characterization of the activities of recombinant AA5_1 and AA5_2 CROs from *V. dahliae* and *V. longisporum*. These data provide critical fundamental information for exploring the biological function of these enzymes and for their potential future application as biocatalysts.

As introduced above, genes encoding a tandem pair of an AA5_2 alcohol oxidase (AlcOx) and a heme-peroxidase (PerOx), which are conserved among foliar phytopathogens from the genera *Colletotrichum* and *Magnaporthe*, have been implicated recently in fungal pathogenesis (23, 24). However, it was unknown how broadly the roles of the enzymes might be conserved across different fungal phytopathogens, including those that employ different modes of infection.

In this study, we showed that genes that encode orthologous AA5_2 enzymes and predicted catalases (from the peroxidase/catalase superfamily) in the vascular wilt pathogens *V. dahliae* JR2 and *V. longisporum* VL43 are indeed arranged in tandem, albeit with a distinct tail-to-tail orientation. The lack of similar expression patterns in different growth conditions (Fig. 4A) and the tail-to-tail arrangement (Fig. 1B) indicate that the *Vda*AA5_2 and catalase genes are not under a common regulator. Additionally, signal peptide analysis predicted that the AA5_2 members should be secreted, while the catalases should be intracellular. In contrast, the genes encoding the AlcOx/PerOx pair in *Colletotrichum,* arranged in a head-to-head orientation, are co-regulated under a suspected bidirectional promoter, and both encode secreted enzymes (24). Although deletion of the gene encoding the AA5_2 AlcOx in *C. orbiculare* and *M. oryzae* significantly reduced pathogenicity (23, 24), reverse genetics of the ortholog in *Vda*AA5_2 did not affect fungal growth, conidiation, or virulence. This suggests that the tandem PerOx-AlcOx infection paradigm in *Colletotrichum* and *Magnaporthe* is not conserved in *Verticillium* species. Similarly, analysis of several public transcriptomic data sets for other *V. dahliae* strains indicated that genes encoding the AA5_2 enzyme and catalase were not similarly expressed post host inoculation or during microsclerotia development (Fig. 4B and C).

With regard to function or activity, a point to consider is the ecological difference between *Verticillium* and *Colletotrichum*, which would influence the substrates accessible to the CROs. The AA5 subfamily 2 (AA5_2) alcohol oxidase in *C. orbiculare* has been shown to oxidize long chain alcohols of the cuticular waxes (24), and while we were unable to identify the natural substrate of *Vda*AA5_2, it was shown to have high activity on aromatic alcohol substrates. As *Verticillium spp*. infiltrate their plant hosts via the root system (4), based on the aryl alcohol oxidase activity of *Vda*AA5_2 (Fig. 3A), putative substrates could include aromatic alcohol units in root structures such as suberin, a biopolymer found in vascular plants (44). Suberin contains polyaliphatic and polyaromatic domains, where the latter has been found to contain lignin-like components such as coniferyl alcohol, sinapyl alcohol, or coumaryl alcohol derived from hydroxycinnamic acids (44, 45). On the other hand, subfamily 1 CROs may be acting as accessory enzymes by oxidizing a broad range of compounds to generate H_2_O_2_ for other enzymes that utilize peroxide. This system has previously been shown in basidiomycetes, such as *Phanerochaete* species, that degrade biomass (21, 46, 47).

Notably, we did observe an apparent slight reduction in pathogenicity following deletion of the AA5_1 gene in *V. dahliae* JR2, evidenced by a larger population of healthy tomato plants post-infection compared to wild-type; however, this difference was not found to be statistically significant. Interestingly, analysis of three different public RNA-seq data sets demonstrated that the AA5_1 gene in *V. dahliae* is differentially expressed following growth in media mimicking plant material (Fig. 4A), post-inoculation of plant host (cotton), and during microsclerotia formation (Fig. 4B and C). Despite the lack of statistical significance in the virulence levels of Δ*Vda*AA5_1, qualitatively, the plant symptoms and transcription patterns highlight the need for further studies in order to reveal the true physiological role of *Vda*AA5_1.

Although biological data are scarce, the potential implication of *Vda*AA5_1 in virulence has analogies in recent studies of the insect and human pathogenic fungi, respectively, *Metarhizium acridum* and *Cryptococcus neoformans*, in which deletion of AA5_1 CRO genes likewise attenuates virulence (39, 48). In these systems, the detoxification of harmful aldehydes, for example, methylglyoxal by AA5_1 CROs, along with the concomitant production of H_2_O_2_, is postulated to promote virulence (39, 48), and it is possible that the aldehyde oxidase activity of *Vda*AA5_1 (Fig. 3C and Table 1) may play similar roles in *V. dahliae*. However, without experimental evidence of the physiological substrate identities or their location of action, it is difficult to surmise the potential cellular functions of the AA5 enzymes in this study. Interestingly, cell wall-associated AA5_1 CROs from the fungi *Ustilago maydis* and *Trichoderma virens* have been reported to be involved in fungal growth, hyphal morphology, and pathogenicity (38, 49). In contrast, deletion of the secreted *Vda*AA5_1 did not affect general growth and conidiation.

Regarding the *V. dahliae* catalase and peroxidases, because we were unable to obtain the recombinant enzymes (*Vda*Catalase, *Vda*PerOx1, and *Vda*PerOx2) or single deletion strains (Δ*Vda*Catalase and Δ*Vda*PerOx2), it is difficult to discuss their putative roles without experimental evidence of their biochemical activity and *in-vivo* or *in planta* effects. Alternatively, looking to bioinformatics (e.g., FungiFun3 server) (50), Gene Ontology (GO), Kyoto Encyclopedia of Genes and Genomes (KEGG), and Functional Catalog (FunCat) functional annotations of the *V. dahliae* catalase and peroxidases report generalized processes such as oxidative stress response, detoxification, and secondary metabolism, etc., which may be involved in a broad range of processes such as regulating defenses against host immune responses or fungal development (51–53).

*Verticillium* fungi typically infect hosts by entering the root system via wound openings and lateral root junctions (9, 54) or through specialized hyphae (i.e., hyphopodia) (11) and the action of cell-wall-degrading enzymes (CWDEs), which belong to the CAZy classification. In comparison to other fungi, the genomes of *Verticillium* spp. are reported to encode a greater number of carbohydrate active enzymes, particularly pectin-degrading enzymes (5, 55). A recent study has shown that the enzymes, *Vd*Cut1 and *Vd*PL16, which belong to the carbohydrate esterase (CE) and polysaccharide lyase (PL) classes, respectively, within CAZy are crucial for root entry and vascular colonization in a hypervirulent *V. dahliae* strain (56). However, the understanding of how the diverse CWDEs found in *V. dahliae* drive pathogenicity is limited by the incomplete functional characterization of these enzymes.

In conclusion, the biochemical characterization of the enzyme activity of three new CROs from *V. dahliae* and *V. longisporum* in this work provides biochemical data that will be useful for enzyme selection for protein engineering efforts. As no statistically significant effects were observed, further studies will clearly be required to fully elucidate the biological roles of CRO homologs in *Verticillium* species, as well as other fungi, to understand why they are widely maintained in genomes. A key challenge in this regard is to fully elucidate the natural substrates of CROs *in vivo*, which is complicated by their demonstrable catalytic promiscuity.

## MATERIALS AND METHODS

### Chemicals and enzymes

All substrate stocks and buffer solutions were prepared with Ultrapure water (18.2 MΩ·cm) unless otherwise stated. All substrates and reagents were purchased from commercial sources (Sigma-Aldrich, VWR, or Fisher) and used without further purification. Horseradish peroxidase (Rz>3.0, BioBasic Canada, Inc.) was used as received.

### Sequence analysis and bioinformatics

All amino acid sequences of AA5 catalytic modules omitting any accessory modules and signal peptides were aligned with the MAFFT algorithm (57). This alignment was used to generate a maximum likelihood phylogenetic tree using the raxmlGUI program (58). All sequence similarity and identity analyses were carried out using BLASTp (59) and amino acid sequences of the AA5 catalytic modules without signal peptides and accessory domains. Signal peptide analyses were conducted using SignalP 6.0 (60).

### Recombinant protein production

cDNA encoding full-length constructs of *Vda*AA5_2 and *Vlo*AA5_2 without signal peptides were commercially synthesized (IDT). cDNA corresponding to a truncated construct of *Vda*AA5_1 containing a single WSC domain was also commercially synthesized (Twist Bioscience). All gene constructs were cloned directly into the pPICZαA vector containing a C-terminal 6× His-tag using the EcoRI and XbaI restriction sites flush with the *Saccharomyces cerevisiae* α-factor signal peptide sequence. The recombinant plasmids were transformed into chemically competent *Escherichia coli* DH5α via heat shock, and recombinant strains were produced as described in the Invitrogen EasySelect Pichia system manuals (Invitrogen). *Komagataella pfaffii* (syn. *Pichia pastoris*) X-33 or KM71H transformants were selected on YPD agar plates containing 100 or 500 µg/mL of Zeocin. The same procedure was followed to generate recombinant strains for the *Vda*PerOx1, *Vda*PerOx2, and *Vda*Catalase constructs.

Large-scale protein production and purification were performed as previously described (32). Protein expression trials for the peroxidase and catalase enzymes were performed as previously described (24, 61). Secreted proteins were purified from supernatant as previously described (32). Proteins were visualized using Coomassie blue R-250; concentrations were determined by measuring *A_280_* using extinction coefficients, and molecular weights were calculated with ProtParam on the ExPASY server (*Vda*AA5_2: 79 kDa, 105,240 M^−1^cm^−1^, *Vlo*AA5_2: 79 kDa, 105,240 M^−1^cm^−1^, and *Vda*AA5_1: 67 kDa, 118,760 M^−1^cm^−1^).

### Enzyme kinetics

Enzyme activity was surveyed on a panel of select substrates using the HRP-ABTS-coupled assay in a reaction volume of 200 µL (50 mM sodium phosphate, pH 7.0, 0.25 mg/mL ABTS, 0.1 mg/mL HRP) in a 96-well plate at room temperature. Absorbance was measured at 415 nm on a BioTek Epoch microplate spectrophotometer. Carbohydrates (galactose, melibiose, lactose, and raffinose) were measured at 300 mM, glycerol at 300 mM, and polysaccharides (arabinan, galactan, galactomannan, and xyloglucan) were measured at 2 mg/mL. Alcohols and aldehydes were screened at 10 mM. Activity across a range of pH values was determined as above, using the HRP-ABTS coupled assay and various buffers to cover a pH range of 5.0–10.5. *Vda*AA5_2 and *Vlo*AA5_2 pH rates were assayed on 300 mM melibiose, while VdaAA5_1 was measured on 10 mM methylglyoxal.

A range of substrate concentrations for a select set of substrates was used to determine the Michaelis-Menten parameters of the target enzymes. The reactions were measured using the HRP-ABTS coupled assay with 0.46 mM ABTS, 21 U/mL HRP in 50 mM sodium phosphate buffers at the previously determined pH optima. Measurements were carried out at 25˚C in 1 mL plastic cuvettes using the Cary 60 UV-VIS spectrophotometer. The Michaelis-Menten equation or a linear fit was applied to the data using OriginPro (OriginLab 9.85).

### *Ex vivo* metabolomics

#### *Arabidopsis thaliana* root cultivation and metabolite extraction

Cultivation of root biomass was conducted as previously described (62), with minor changes. Briefly, *A. thaliana* seeds were surface sterilized with chlorine gas at room temperature and resuspended in 50 μL sterile water. Seeds (~20) were spread onto sterile support matrix screens and placed onto 0.5% Murashige and Skoog (MS) media nutrient agar plates (0.5× MS with B5 vitamins, 25 mg/L MES, 0.7% agar, pH 5.8). The plates were placed under 16 h day fluorescent light conditions for 7 days at 22°C. After germination of the seeds, the seedlings were transferred into sterile flasks containing 10 mL of 0.5% MS media with 1% sucrose and incubated at room temperature on a rotary shaker (70 rpm) for 2 days under 16 h light conditions. After 2 days, the seedlings were placed in fresh media and incubated for another 2 days. The seedlings were transferred into 0.5% MS media with 3% sucrose and incubated for a week; the media were changed two more times during the week. Following this, the seedlings were transferred into 0.5% MS media with no sucrose and incubated for 3 days, then placed into fresh media for another 2 days, after which the root mass was harvested and immediately frozen in liquid nitrogen.

Before extraction, plant material was kept freeze-dried and ground using a Mixer Ball Mill MM200 until it was homogenized into a powder. The workflow followed a two-phase extraction method described in a previous study (63). Polar and non-polar metabolites were extracted with methyl-tert-butyl ether (MTBE), methanol, and water. The collected phases were transferred into a Kimble glass tube and dried with a stream of nitrogen. The samples were resolubilized in methanol, transferred to a reaction tube, and dried again, after which the samples were solubilized in 10 mM HEPES buffer, pH 7.0.

### Enzyme addition and UHPLC-HRMS analysis

Enzymatic reactions were set up in a total volume of 1 mL containing 150 μL of root extract with 0.5 mg/mL of both horseradish peroxidase and catalase. Reactions were initiated with up to 1 mg/mL of enzyme and incubated for 22 h or 3 days at 30°C. Enzymes were then inactivated by the addition of 1 mL of acetonitrile, centrifuged for 5 min at 18,000 × *g* and 4°C, and transferred to LC-MS vials.

To identify substrate(s) and product(s) from the enzymatic assay of the root extracts, samples were analyzed in both positive and negative ionization modes. *Ex vivo* extracts were analyzed using a 1290 UHPLC (Agilent Technologies) coupled to a PDA detector and the 6540 UHD Accurate-Mass Q-TOF LC-MS instrument with the Agilent Dual Jet Stream Technology as an ESI source. In addition, an isocratic pump was included to infuse the reference solution in parallel for automated mass correction (64). The system was equipped with an ACQUITY HSS T3 column (2.1 × 100 mm, 1.8 µm particle size, Waters Corporation). A solvent gradient of water (A) to acetonitrile (B), both supplemented with 0.1% formic acid, was run according to parameters as described in a previous study (63). For targeted MS-MS fragmentation, exact precursor ion masses and retention times were used to select individual compounds by a quadrupole. The fragmentation was achieved by collision-induced dissociation with inert N_2_ gas. Collision energy was set between 10 and 40 eV for optimized fragmentation, depending on the compound.

Various databases were used for the interpretation of fragmentation data. METLIN, MassBank, NIST MS, as well as in-house databases were used for the identification of potential compound hits. If in the case, no hits are found, SIRIUS (65) was used to generate putative structures. For data analysis, the Mass Hunter Workstation Qualitative Analysis 10.0 (Agilent) and Profinder B08.00 software were used. Data processing and visualization were carried out using the MarVis toolbox (MarVis Cluster, Filter, and Pathway ver 2.6) (66–68).

### Reverse genetics

The *VdaAA5_2* (*VDAG_JR2_Chr6g10290a*), *VdaAA5_1* (*VDAG_JR2_Chr3g06330a*), and *Perox1* (*VDAG_JR2_Chr2g10970*) genes from *V. dahliae* were replaced by resistance marker (hygromycin or nourseothricin) cassettes via homologous recombination. Single deletion strains were generated. DNA fragments were amplified by PCR with Phusion DNA polymerases (Thermo Fisher Scientific) and purified using the NucleoSpin Gel and PCR clean-up kit (Macherey-Nagel). Primers were designed with a minimum of 15 base pairs of homology to the desired neighboring sequences to allow assembly of the DNA fragments using the GeneArt Seamless Cloning and Assembly kit (Thermo Fisher Scientific). The resulting plasmids were transformed into *E. coli* DH5α via heat shock, and successful transformants were confirmed by sequencing (Microsynth Seqlab, Göttingen, Germany). *Agrobacterium tumefaciens* AGL1 was transformed as previously described (69); successful transformants were selected with 100 µg/mL kanamycin and confirmed by colony PCR. Confirmed *A. tumefaciens* transformants were then used to transform *V. dahliae*. All primers, plasmids, and bacterial or fungal strains used in this study are listed in Tables S2 and S3.

For isolation of genomic DNA, *V. dahliae* strains were grown at 25˚C in liquid potato dextrose medium (PDM) with shaking, 120 rpm, for 5–7 days. Mycelia were harvested using Miracloth and ground into powder in liquid nitrogen. Genomic DNA was isolated with phenol, as described previously (55). Southern hybridizations were performed using Amersham AlkPhos Direct Labeling and CDP-Star detection reagents (GE Healthcare).

The *VdaAA5_1* complementation strain was constructed by ectopically reintroducing the *VdaAA5_1* gene into the *VdaAA5_1* deletion strain. The *VdaAA5_1* gene and its native promoter were amplified from the *V. dahliae* JR2 WT genome by PCR. The amplified *VdaAA5_1* complementation cassette and the nourseothricin resistance marker were assembled into a plasmid, according to the methods described above. The assembled plasmid was transformed into *E. coli* DH5α, and the plasmid was confirmed by sequencing. The *VdaAA5_1* complementation cassette was ectopically integrated into the genome of the *V. dahliae VdaAA5_1* deletion strain by *Agrobacterium*-mediated transformation. The presence of *VdaAA5_1* in the transformant genome was confirmed by Southern hybridization.

### Phenotyping

Colony growth and microsclerotia formation on media containing 2% agar were studied after point inoculation of 5 × 10^4^ spores and incubation at 25˚C for 10 days. PDM, SXM, and CDM were used. Morphology under different stress conditions for each deletion strain was tested by replacing sucrose in CDM with 3% cellulose, or galactose, or supplementing with 0.8 M sorbitol, 0.5 M NaCl, 0.004% SDS, 7.5 × 10^−5^% H_2_O,_2,_ 0.5 mM CuSO4, or 5% ethanol (vol/vol).

*Solanum lycopersicum* (“Moneymaker”) seeds were surface sterilized with 70% (vol/vol) ethanol and 0.05% Tween20. Ten-day-old seedlings were wounded and inoculated with spores or water by root dipping as previously described (70). After 21 days of incubation under long-day conditions in a BrightBoy GroBank (CLF Plant Climatics, Germany), discoloration of tomato hypocotyls was observed with binocular microscopy. Total disease scores of tomato plants are presented in stacking diagrams. Disease symptoms were determined by measuring plant weight, height, and the length of the longest leaf. These measures were converted to a disease score ranking relative to the mean values of mock-inoculated plants (set to 100%). Plants were classified as displaying healthy (>80%), mild symptoms (60%–80%), strong symptoms (40%–60%), and very strong symptoms (<40%). Fifteen or more biological replicates (*n* = >30 plants) were measured for each infection set.

## Data Availability

Data generated and analyzed during this study are included in this article and the associated supplemental material. All nucleotide sequences, protein sequences, and protein structural information used in this work were extracted from public databases, that is, GenBank (https://www.ncbi.nlm.nih.gov/genbank/), The Protein Data Bank (https://www.rcsb.org/), The CAZy database (https://www.cazy.org/), JGI Mycocosm (mycocosm.jgi.doe.gov/mycocosm/home), and EnsemblFungi (https://fungi.ensembl.org/). Public RNA-seq data sets were accessed from the NCBI (BioProject IDs PRJNA1346639, PRJNA1364727, PRJNA208120, and PRJNA1219802).

## SUPPLEMENTAL MATERIAL

Supplemental material aem.00790-26-s0001.pdf Tables S1 to S3; Fig. S1 to S8.

ASM does not own the copyrights to Supplemental Material that may be linked to, or accessed through, an article. The authors have granted ASM a non-exclusive, world-wide license to publish the Supplemental Material files. Please contact the corresponding author directly for reuse.
