# Supplementary material for: Exploring the biochemical and biological functions of copper radical oxidases in the vascular wilt phytopathogen Verticillium dahliae
Source: Appl Environ Microbiol. 2026 Jun 3;92(7):e00790-26. doi: 10.1128/aem.00790-26 (PMC13390370; doi:10.1128/aem.00790-26)
Supplement: Supplemental material — Tables S1 to S3; Fig. S1 to S8. [file aem.00790-26-s0001.pdf]

Exploring the biochemical and biological functions of Copper Radical Oxidases in the vascular wilt phytopathogen *Verticillium dahliae*

Jessica K Fong, Ying-Yu Chen, Rebekka Harting, Moritz Klein, Simone Lewandowski, Yann Mathieu, Mireille Haon, Bastien Bissaro, Jean-Guy Berrin, Ivo Feussner, Gerhard H. Braus, Harry Brumer*

Supplementary Information

Supplementary Tables

Table S1. Specific activities values for CROs from *Verticillium dahliae.*

Enzyme	Substrates	Specific Activity (µmol/min/mg)
*Vda*AA5_2	Furfuryl alcohol	$(15.0 \pm 0.6) \times 10^{-1}$
	Cinnamyl alcohol	$(15.0 \pm 0.8) \times 10^{-1}$
	Hydroxymethyl furfural	$(30.0 \pm 0.5) \times 10^{-1}$
	Benzyl alcohol	$(18.0 \pm 2.6) \times 10^{-1}$
	1,3 - Propanediol	$(14.0 \pm 0.1) \times 10^{-2}$
	Glycerol	$(27.0 \pm 0.7) \times 10^{-1}$
	Xyloglucan	$(2.0 \pm 0.5) \times 10^{-2}$
	Galactomannan	$(2.0 \pm 0.5) \times 10^{-2}$
	Arabinan	$(19.0 \pm 0.1) \times 10^{-1}$
	Raffinose	$(33.0 \pm 0.9) \times 10^{-1}$
	Melibiose	$(40.0 \pm 1.2) \times 10^{-1}$
	Lactose	$(5.9 \pm 0.6) \times 10^{-1}$
	Arabinose	$(36.0 \pm 0.7) \times 10^{-2}$
	Xylose	$(29.0 \pm 0.1) \times 10^{-2}$
	Galactose	$(8.0 \pm 0.2) \times 10^{-1}$
*Vlo*AA5_2	Furfuryl alcohol	$(3.3 \pm 0.1) \times 10^{-1}$
	Cinnamyl alcohol	$(5.2 \pm 0.3) \times 10^{-1}$
	Hydroxymethyl furfural	$(8.8 \pm 0.5) \times 10^{-1}$
	Benzyl alcohol	$(4.7 \pm 0.2) \times 10^{-1}$
	1,3 - Propanediol	$(0.4 \pm 0.1) \times 10^{-1}$
	Glycerol	$(8.7 \pm 0.2) \times 10^{-1}$
	Xyloglucan	$(2.0 \pm 0.1) \times 10^{-2}$
	Galactan	$(9.0 \pm 0.8) \times 10^{-3}$
	Galactomannan	$(10.0 \pm 0.8) \times 10^{-3}$
	Arabinan	$(6.6 \pm 0.4) \times 10^{-1}$
	Raffinose	$(6.5 \pm 0.4) \times 10^{-1}$
	Melibiose	$(11.0 \pm 0.6) \times 10^{-1}$
	Xylose	$(2.0 \pm 0.1) \times 10^{-1}$
	Lactose	$(23.0 \pm 0.6) \times 10^{-2}$
	Galactose	$(29.0 \pm 0.3) \times 10^{-2}$
*Vda*AA5_1	D – glyceraldehyde	5.0 ± 1.0
	L - glyceraldehyde	$(0.4 \pm 0.1) \times 10^{-1}$
	Methyl glyoxal	3.0 ± 0.2
	Glyoxal	$(0.6 \pm 0.1) \times 10^{-1}$
	Galactose	2.5 ± 0.1
	Glycerol	1.0 ± 0.1

*HRP-ABTS coupled assay in 50 mM sodium phosphate buffer, pH 7.0 at room temperature with 300 mM carbohydrates or polyols, 10 mM alcohol or aldehyde substrates and 2mg/mL polysaccharides.

Table S2. List of primers used in this study for construction of *V. dahliae* JR2 strains

Primers	Sequence (5' – 3')	Overhang to	Description
ML8	AAAGAAGGATTACCTCTAAACAA	n/a	For resistance marker
ML9	TGTACAGTGACCGGTGAC	n/a	
AN118	CCGCGACGTTAACTGATATTG	n/a	
YYC35	CCGCGACGTTAACTGATATTGA	n/a	
RH898	ATTCTTAATTAAGATTGACGAGCAACACAGACA	pSAB12	For *Vda*AA5_2 5' and 3' flanking regions
RH899	ACCGGTCACTGTACAGTTGAGAATTGAGAGTTTGG	^{p}*gpdA*	
RH900	AGGACTTCTAGAAGGTAGATGCTATCTTCTCAGAA	pSAB12	
RH901	AGGTAATCCTTCTTTACAGATCATCACAACTTGTC	*trpC*^{t}	
RH906	ATTCTTAATTAAGATTCGCTGTGAGAGCTTTCAG	pSAB12	For *Vda*PerOx1 5' and 3' flanking regions
RH907	ACCGGTCACTGTACAGCTGCAAATGAGGTACTAAG	^{p}*gpdA*	
RH908	AGGTAATCCTTCTTTTGGAGAGAGGATTCTGCAAT	*trpC*^{t}	
RH909	AGGACTTCTAGAAGGCCATTTTGGGATAGAGTCGA	pSAB12	
RH956	ATTCTTAATTAAGATTCATCAGGGGGATCATCATG	pKO2-11	For *Vda*AA5_1 5' and 3' flanking regions
RH957	CAGTTAACGTCGCGGGTTGACTGTTGTTTGCTAGG	^{p}*trpC*	
RH958	AGGTAATCCTTCTTTAGTTGAACTGATGGATTTCTC	*trpC*^{t}	
RH959	AGGACTTCTAGAAGGATGCGGGATTAGCTCGTC	pKO2-11	
YYC107	CAGTTAACGTCGCGGCTACAAGCCAGGTGGTGAAAAG	^{p}*trpC*	For *Vda*AA5_1 gene

^{p}: promoter, ^{t}: terminator

Table S3. List of plasmids and strains used for the construction of *V. dahliae* JR2 strains

Component	Description
**Plasmids**	
pSAB12	Cloning vector with *KAN*^{R} and *NATR*; left and right border for *A. tumefaciens*-mediated transformation (ATMT)
pKO2-11	Cloning vector with *KAN*^{R} and *HYG*^{R}; left and right border for ATMT
**Strains**	
AGL-1	Strain for *A. tumefaciens* mediated transformation of *V. dahliae*
DH5a	Strain for cloning and extraction of plasmids
JR2/WT	*Solanum lycopersicum* isolate
Δ*VdaAA5_2*	*Vda*AA5_2 deletion strain (Δ*VdaAA5_2::*^{p}*gdpA:NAT*^{R}*:trpC*^{t})
Δ*PerOx1*	PerOx1 deletion strain (Δ*PerOx1::*^{p}*gdpA:NAT*^{R}*:trpC*^{t})
Δ*VdaAA5_1*	*Vda*AA5_1 deletion strain (Δ*VdaAA5_1::*^{p}*trpC:HYG*^{R}*:trpC*^{t})
VdaAA5_1-C	*Vda*AA5_1 complementation strain (Δ*VdaAA5_1::*^{p}*trpC:HYG*^{R}*:trpC*^{t}; ^{p}*VdaAA5_1:VdaAA5_1:*^{p}*trpC:NAT*^{R}*:trpC*^{t})

^{p}: promoter, ^{t}: terminator, *NAT*^{R}: nourseothricin resistance marker, *HYG*^{R}: hygromycin resistance marker.

Supplementary Figures

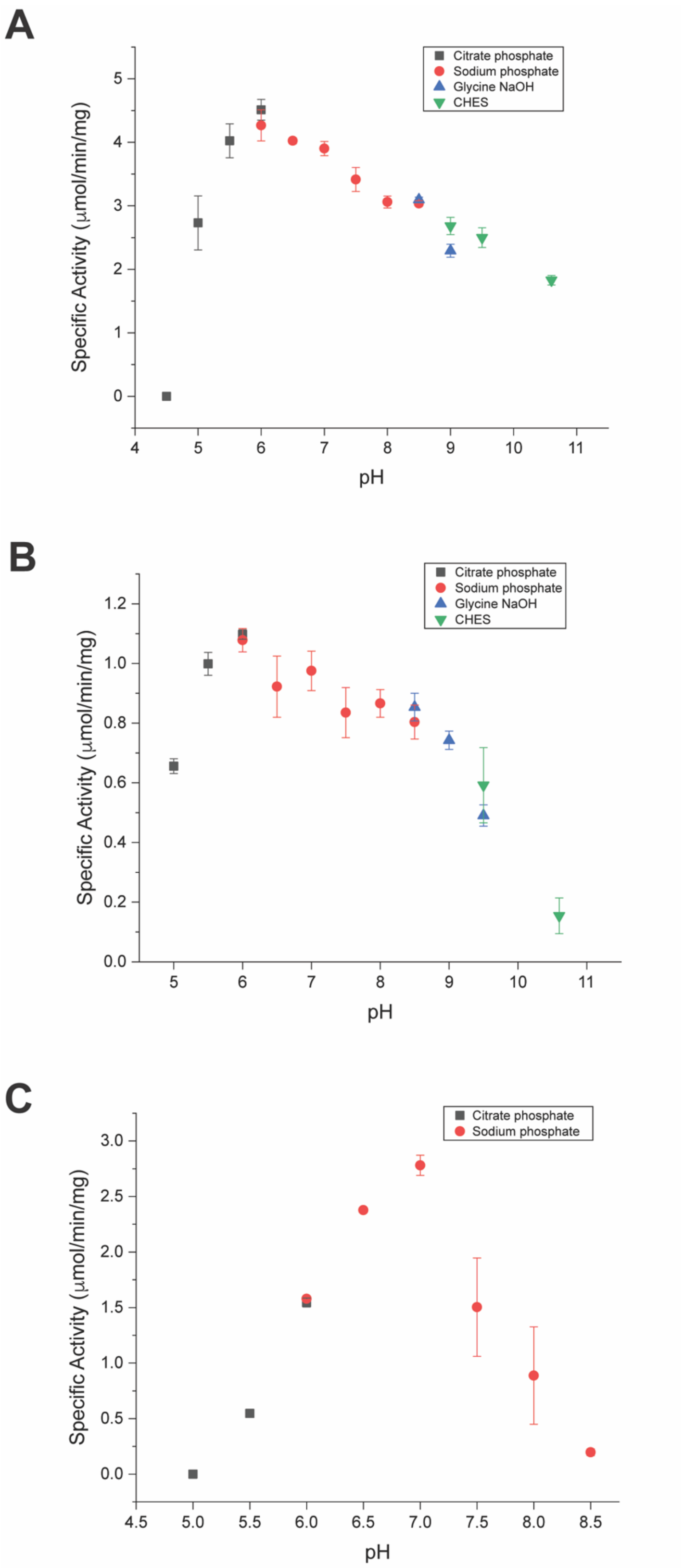

Figure S1. pH – rate profiles of (A) *Vda*AA5_2, (B) *Vlo*AA5_2 and (C) *Vda*AA5_1. pH-rate profiles were measured using the HRP-ABTS coupled assay with 300 mM melibiose, 10 mM methylglyoxal or 300 mM glycerol. Measurements were performed in triplicate at room temperature. Citrate phosphate (black square), sodium phosphate (red circle), glycine NaOH (blue triangle) and CHES (green triangle) buffers were used for a coverage of pH 4.5 – 10.5.

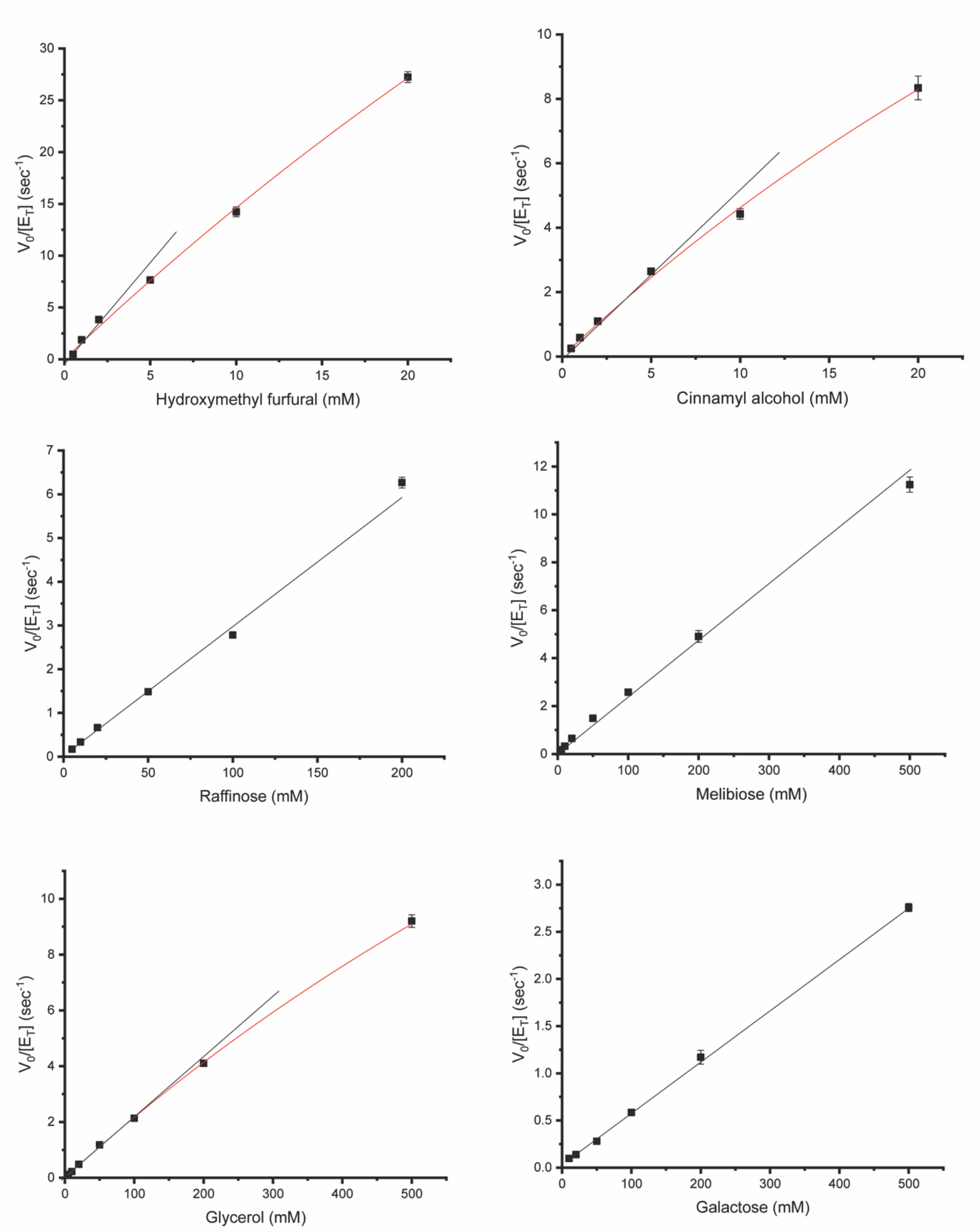

Figure S2. *Vda*AA5_2 Michaelis-Menten kinetics. Initial rate values were measured in triplicate at each substrate concentration. Individual k_{cat} and K_M values were calculated by performing a non-linear fitting analysis of the standard Michaelis-Menten equation (red) using OriginLab 9.85. A linear fit (black) was also applied to data that did not reach saturation.

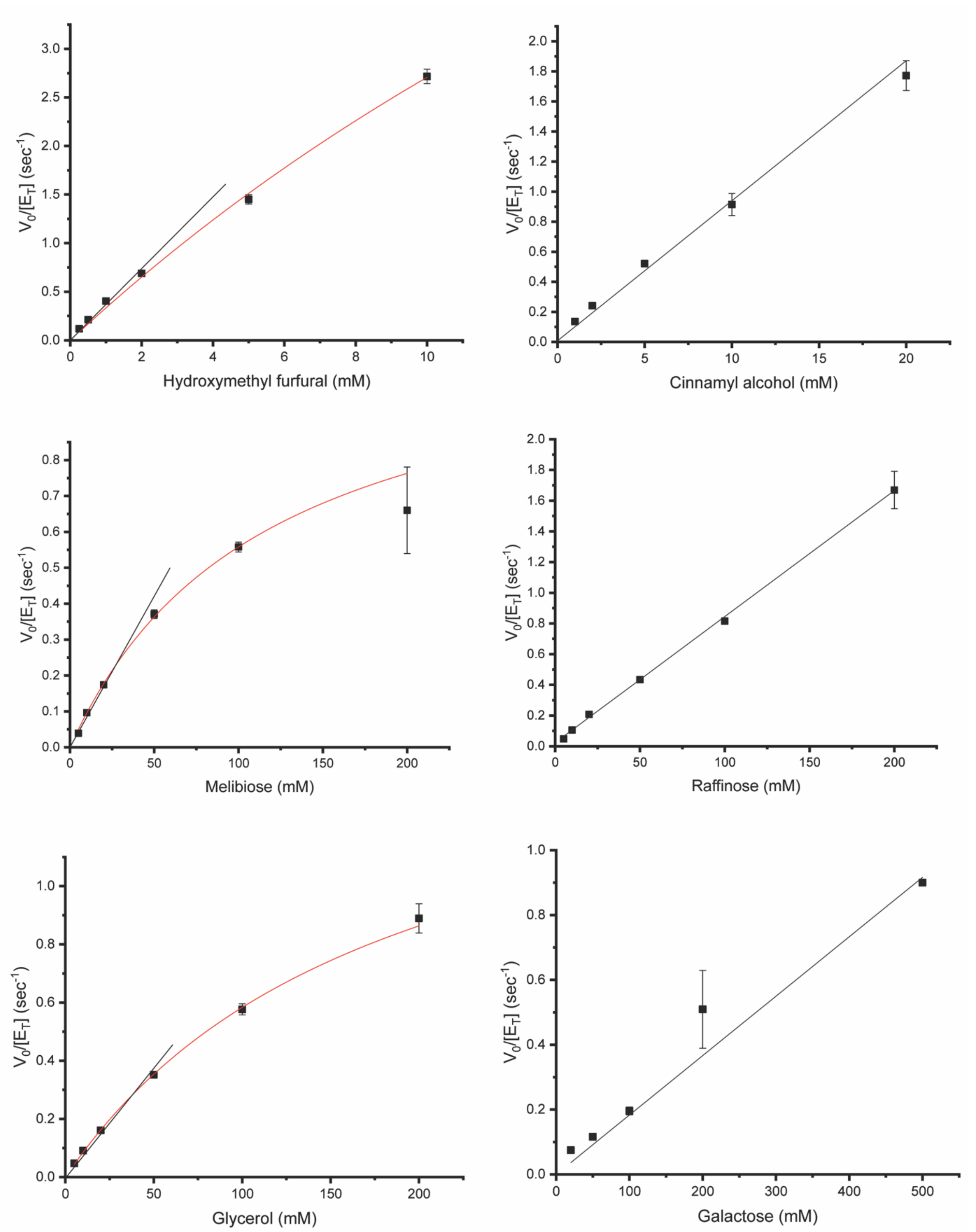

Figure S3. *Vlo*AA5_2 Michaelis-Menten kinetics. Initial rate values were measured in triplicate at each substrate concentration. Individual k_{cat} and K_M values were calculated by performing a non-linear fit of the standard Michaelis-Menten equation (red) using OriginLab 9.85. A linear fit (black) was also applied to data from reactions that did not reach saturation.

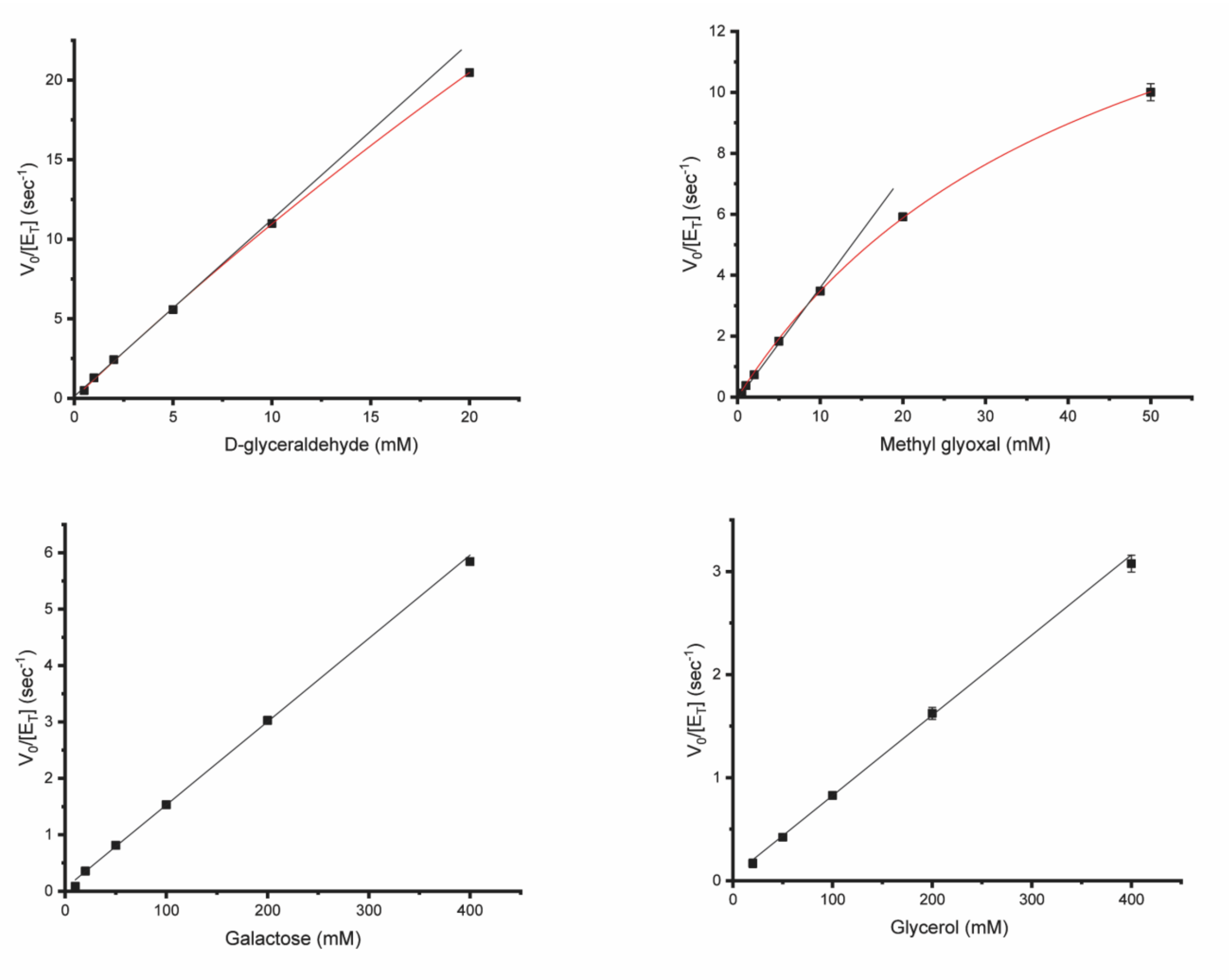

Figure S4. *Vda*AA5_1 Michaelis-Menten kinetics. Initial rate values were measured in triplicate at each substrate concentration. Individual k_{cat} and K_M values were calculated by performing a non-linear fit of the standard Michaelis-Menten equation (red) using OriginLab 9.85. A linear fit (black) was also applied to data from reactions that did not reach saturation.

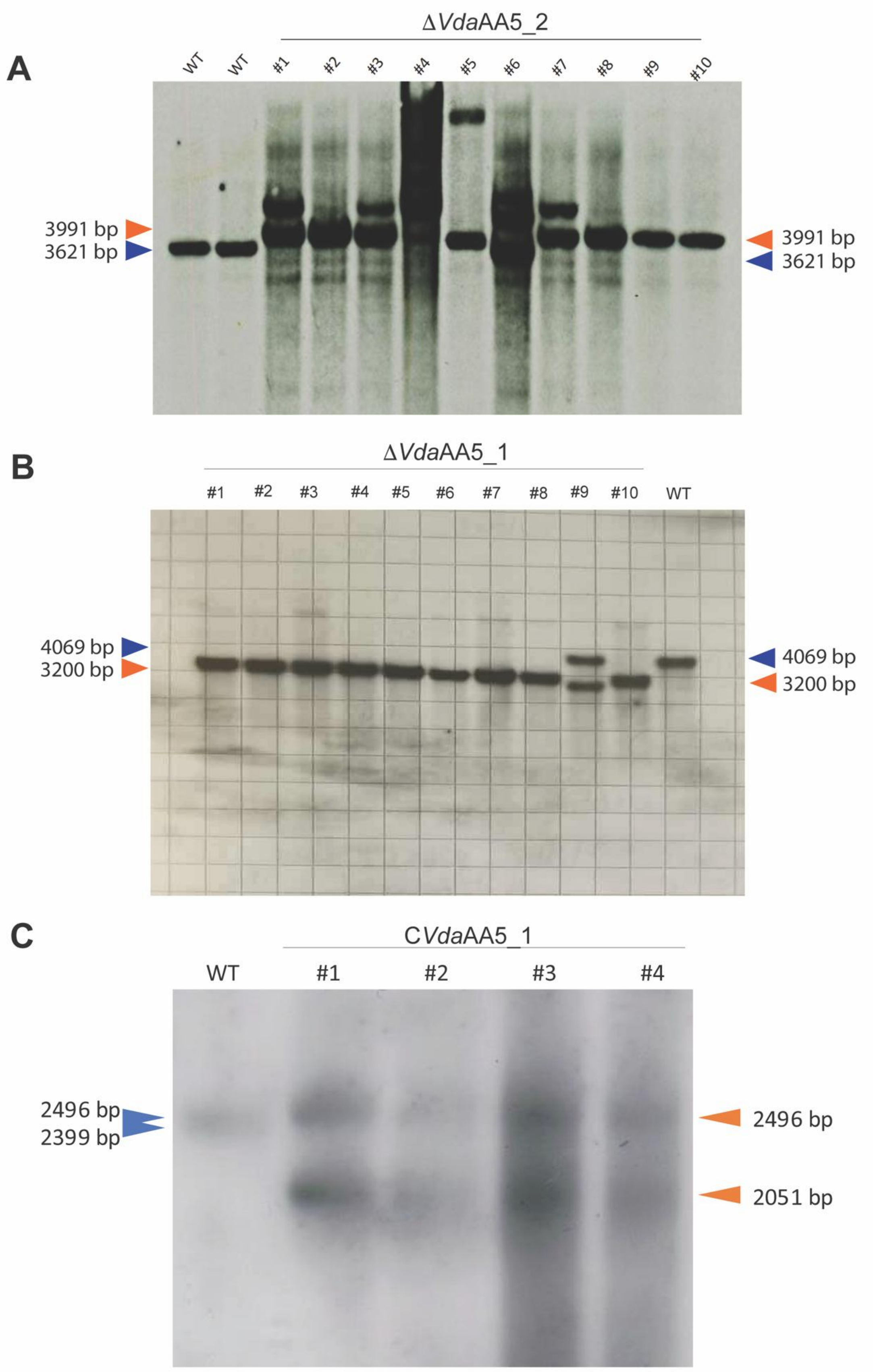
A
ΔVdaAA5_2
WT
WT
#1
#2
#3
#4
#5
#6
#7
#8
#9
#10
3991 bp
3621 bp
3991 bp
3621 bp
B
ΔVdaAA5_1
#1
#2
#3
#4
#5
#6
#7
#8
#9
#10
WT
4069 bp
3200 bp
4069 bp
3200 bp
C
CVdaAA5_1
WT
#1
#2
#3
#4
2496 bp
2399 bp
2496 bp
2051 bp

Figure S5. Southern hybridization verification of *V. dahliae* JR2 deletion or complementation strains. (A) *Vda*AA5_2 deletion verified via digestion with *Nru*I and the 3' flanking region of the gene as a probe (B) *Vda*AA5_1 deletion verified by digestion with *Pst*I and the 3' flanking region as a probe. (C) Complementation of the *Vda*AA5_1 gene was verified via digestion with *Bam*HI and 1803 bp at the 3' end of the gene as a probe. Genomic DNA of wild-type strain (WT) was used as a control. Sizes of expected fragments are indicated in blue (wildtype) and orange (positive clone). A 1kb DNA ladder was used for estimation of fragment size.

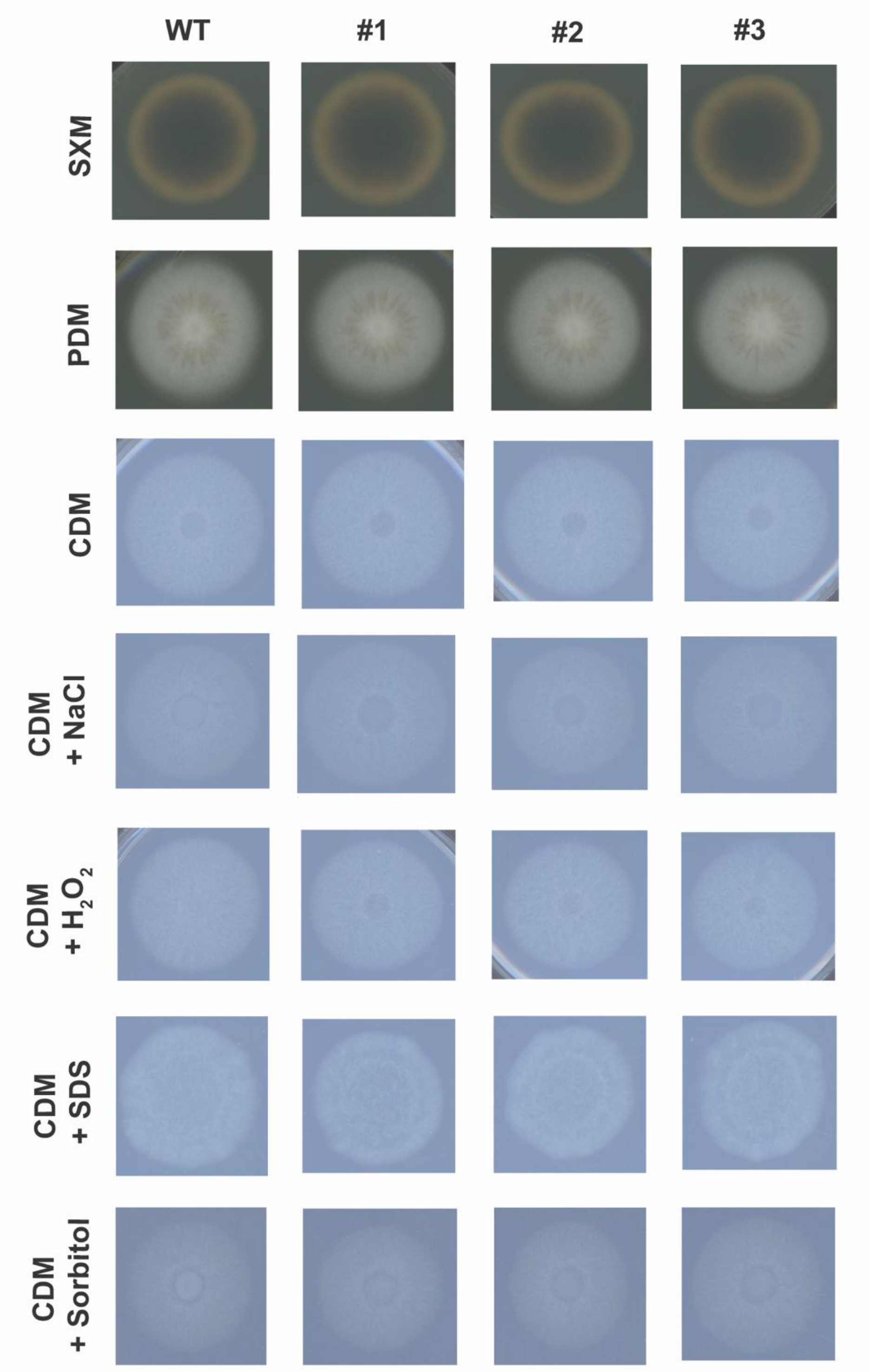

Figure S6. Effects of various stress conditions on the phenotype of the *Vda*AA5_2 deletion strain (Δ*Vda*AA5_2). Fungal colonies were spot inoculated with spores of the *V. dahliae* wild-type strain (WT) and individual clones of the Δ*Vda*AA5_2 strain (#1-3), then incubated at 25˚C for 10 days. All photos were imaged from a top view, except for the SXM agar medium.

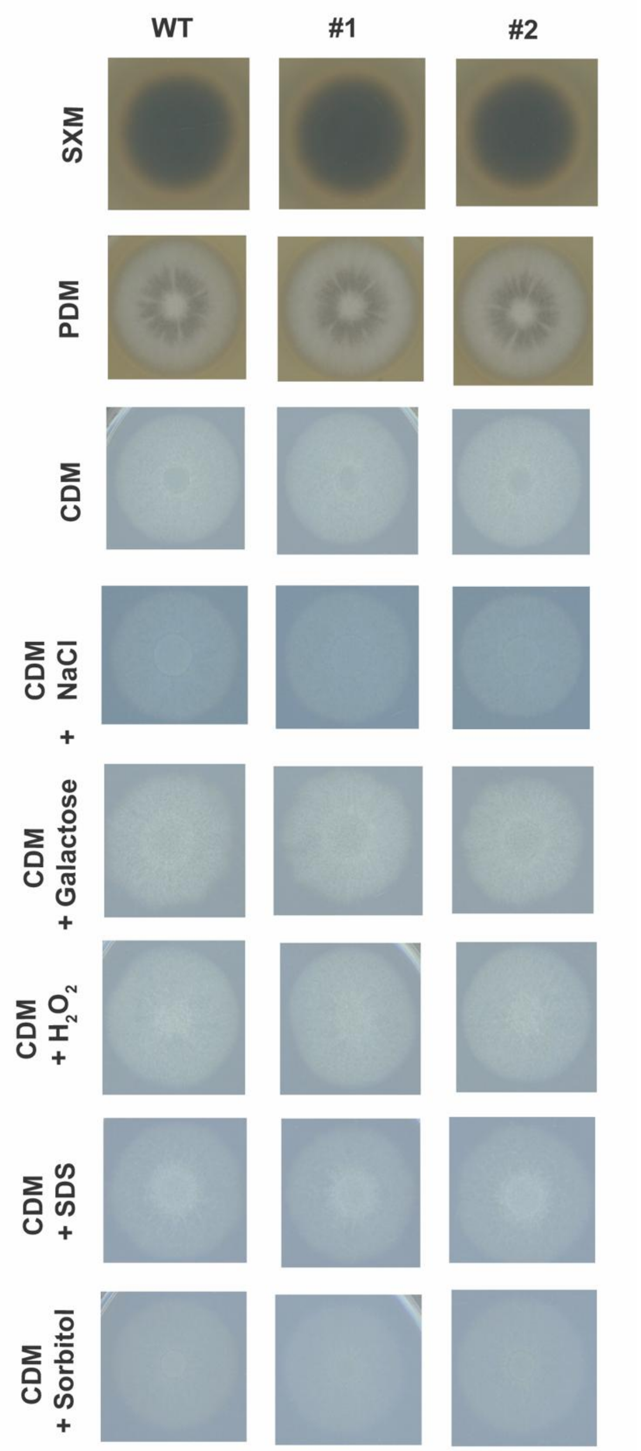

Figure S7. Effects of various stress conditions on the phenotype of the *Vda*PerOx1 deletion strain (Δ*Vda*PerOx1). Fungal colonies were spot inoculated with spores of the *V. dahliae* wild-type strain (WT) and individual clones of the Δ*Vda*PerOx1 strain (#1-2), then incubated at 25˚C for 10 days. All photos were imaged from a top view, except for the SXM agar medium.

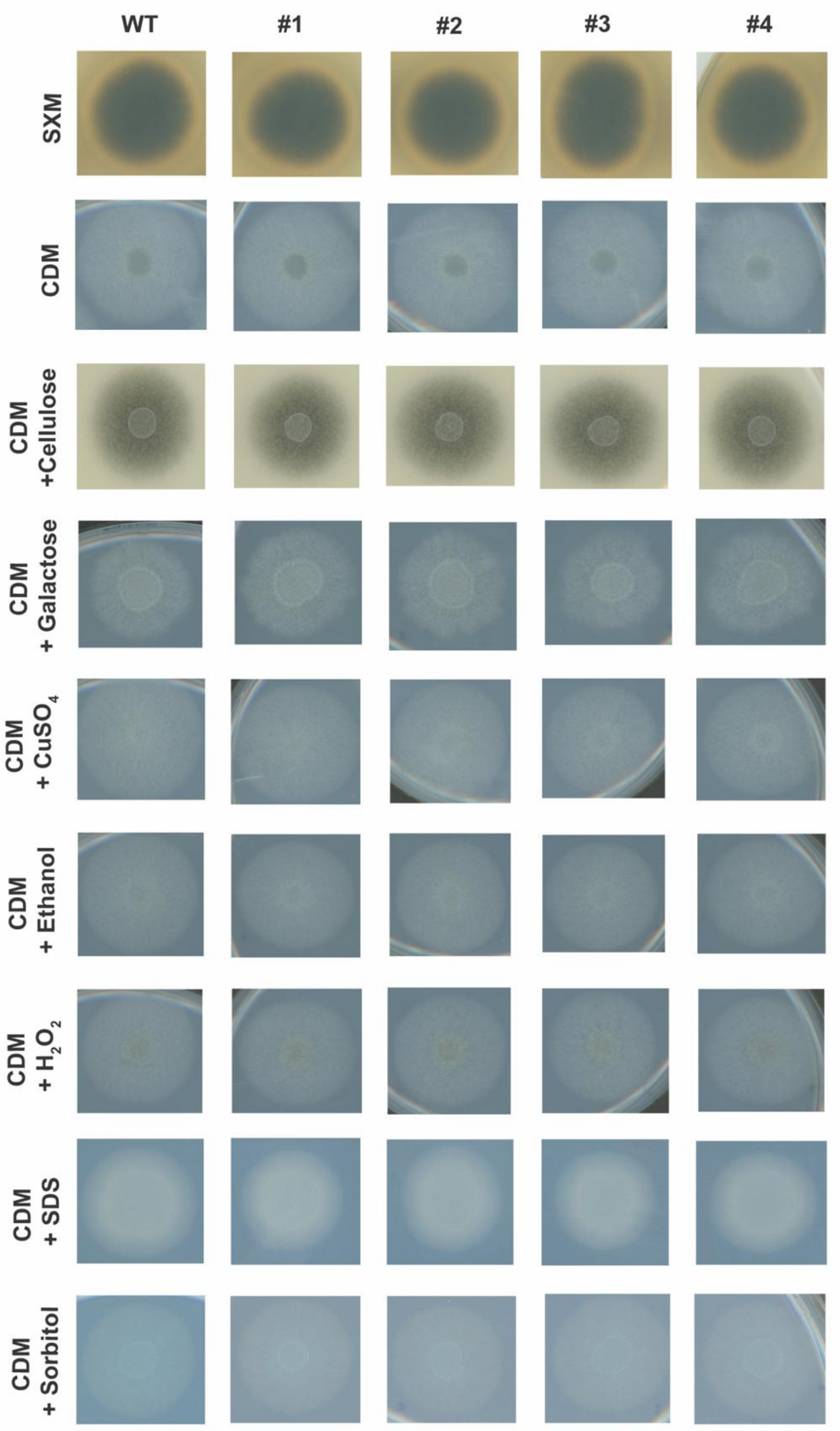

Figure S8. Effects of various stress conditions on the phenotype of the *Vda*AA5_1 deletion strain (Δ*Vda*AA5_1). Fungal colonies were spot inoculated with spores of the *V. dahliae* wild-type strain (WT) and individual clones of the Δ*Vda*GlyOx strain (#1-4), then incubated at 25˚C for 10 days. All photos were imaged from a top view, except for the SXM agar medium.
